# Fibromyalgia and Shoulder Surgery: A Systematic Review and a Critical Appraisal of the Literature

**DOI:** 10.3390/jcm8101518

**Published:** 2019-09-21

**Authors:** Riccardo Compagnoni, Roberta Gualtierotti, Francesco Luceri, Fabio Sciancalepore, Pietro Simone Randelli

**Affiliations:** 1 Laboratory of Applied Biomechanics, Department of Biomedical Sciences for Health, Università degli Studi di Milano, Via Mangiagalli 31, 20133 Milan, Italy; riccardo.compagnoni@gmail.com (R.C.); pietro.randelli@unimi.it (P.S.R.); 2 1° Clinica Ortopedica, ASST Centro Specialistico Ortopedico Traumatologico Gaetano Pini-CTO, Piazza Cardinal Ferrari 1, 20122 Milan, Italy; francesco.luceri@unimi.it (F.L.); fabio.sciancalepore@unimi.it (F.S.); 3 Department of Medical Biotechnology and Translational Medicine, Università degli Studi di Milano, 20090 Milano, Italy

**Keywords:** fibromyalgia, shoulder, surgery, arthroscopy, arthroplasty

## Abstract

Fibromyalgia is a common musculoskeletal syndrome characterized by chronic widespread pain and other systemic manifestations, which has demonstrated a contribution to higher postoperative analgesic consumption to other surgeries such as hysterectomies and knee and hip replacements. The aim of this review is to search current literature for studies considering the impact of fibromyalgia on clinical outcomes of patients undergoing shoulder surgery. A systematic literature review was conducted in PubMed/Medline, Embase, and ClinicalTrials.gov in February 2019. Studies were selected based on the following participants, interventions, comparisons, outcomes, and study design criteria: adult patients undergoing surgery for shoulder pain (P); diagnosis of fibromyalgia (I); patients without fibromyalgia (C); outcome of surgery in terms of pain or analgesic or non-steroidal anti-inflammatory drugs consumption (O); case series, retrospective studies, observational studies, open-label studies, randomized clinical trials, systematic reviews and meta-analyses were included (S). Authors found 678 articles, of which four were found eligible. One retrospective study showed that patients with fibromyalgia had worse clinical postoperative outcomes; two retrospective studies reported a higher opioid prescription in patients with fibromyalgia and one prospective observational study found that a higher fibromyalgia survey score correlated with lower quality of recovery scores two days after surgery. The scarce and low-quality evidence available does not allow confirming that fibromyalgia has an impact on postoperative outcomes in shoulder surgery. Future studies specifically focusing on shoulder surgery outcomes may help improvement and personalization of the management of patients with fibromyalgia syndrome (PROSPERO 2019, CRD42019121180).

## 1. Introduction

Fibromyalgia is a musculoskeletal syndrome characterized by chronic widespread pain and systemic manifestations such as fatigue, non-refreshed sleep, mood disturbance, and cognitive impairment, causing a high impact on health-related quality of life (HRQoL) [1,2]. Although the prevalence in the general population is approximately 3%, ranging from 0.4% to 9.3% worldwide, fibromyalgia is often misdiagnosed, with patients waiting up to two years for a definitive diagnosis [3,4]. The pathogenesis of fibromyalgia seems to be due to the complex interaction of the “central sensitization” with the “peripheral sensitization”. The “central sensitization” is the increased responsiveness of neurons of the central nociceptive pathways by low-threshold, non-noxious inputs such as pressure and temperature [5,6]. The “peripheral sensitization” is the reduction of threshold and the increase in responsiveness of the peripheral nociceptors probably associated with a reduction in epidermal small-fiber density [7,8]. Consequently, patients with fibromyalgia report more pain than normally expected based on the type or degree of stimulus [2,5].

The diagnosis of fibromyalgia is not straightforward because physical examination in these patients is often non-specific, apart from diffuse tenderness. A relevant limit is that actually no specific instrumental test for fibromyalgia is validated and used in clinical practice. Therefore, classification criteria based on history or physical examination have been developed (Table 1) [9,10].

The treatment of fibromyalgia includes a multidisciplinary approach, based on individual needs [11].

Although the most common complaint of patients with fibromyalgia is “pain all over the body”, some patients may refer to the orthopedic surgeon for pain localized in a specific region. Shoulder pain is reported to be one of the most frequent localizations in fibromyalgia, after back pain and neck pain [12].

Fibromyalgia evaluation scales are not routinely assessed by orthopedic surgeons, with a high risk of misdiagnosis of this pathology, which may even coexist in patients with other pathological conditions such as cuff tears or osteoarthritis [13,14]. Medical or surgical options are often performed in these patients, with the risk of unexplained unsatisfactory results, considering that several studies found that fibromyalgia patients may require different postoperative pain management [15,16,17]. A higher opioid consumption in these patients undergoing lower-extremity joint replacement has been demonstrated [15,18], but to date there is a lack of knowledge about the effects on upper limb orthopedic surgery [19,20]. 

The aim of this systematic review is to search the currently available literature regarding the influence of fibromyalgia on clinical outcomes of patients undergoing surgery for shoulder pain.

## 2. Materials and Methods

A systematic literature review was performed from November 2018 to February 2019 without a time limit following the PRISMA statement for transparent reporting of systematic reviews and meta-analyses [21]. The following participants, interventions, comparisons, outcomes, and study design (PICOS) were used: adult patients undergoing surgery for shoulder pain (P); diagnosis of fibromyalgia (I); patients without fibromyalgia (C); outcome of surgery in terms of pain or analgesic or non-steroidal anti-inflammatory drugs (NSAID) consumption (O); case series, retrospective studies, observational studies, open-label studies, randomized clinical trials. Systematic reviews and meta-analyses were included to extract primitive studies (S).

Database search included PubMed/Medline, Embase, and ClinicalTrials.gov. Only articles written in English were selected. PubMed database was searched for the terms (“fibromyalgia” [MeSH Terms] OR “fibromyalgia” [All Fields]) AND (“shoulder” [MeSH Terms] OR “shoulder” [All Fields]). Embase database was searched for the terms (“fibromyalgia”/exp OR fibromyalgia) AND (“shoulder”/exp OR shoulder). ClinicalTrials.gov was searched for the terms “fibromyalgia” AND “shoulder”. Exclusion criteria applied in this review were articles in a different language other than English, case reports, commentaries, letters to the editor and biomechanical studies. All studies were analyzed using title and abstract, and then the full text was collected to establish the coherence with the PICOS of this review. These steps were performed by two different authors (RG and FL) and controversies were resolved after discussion. When an agreement was not achieved, a third researcher (RC) was involved to give a final unanimous selection.

A bias assessment was performed according to the Cochrane handbook guidelines to give a clear strength of clinical indications of this review (allocation, blinding, attrition, reporting, and other potential sources of bias) in selected randomized controlled trials, if available [22]. For all other studies, the free software Review Manager (RevMan 5.3) was used to asses all these biases. In particular, for non-randomized observational studies, we used the Newcastle–Ottawa quality assessment scale [23]. The protocol has been submitted to the PROSPERO registry (https://www.crd.york.ac.uk/prospero, registration number CRD42019121180).

## 3. Results

**Figure 1:**
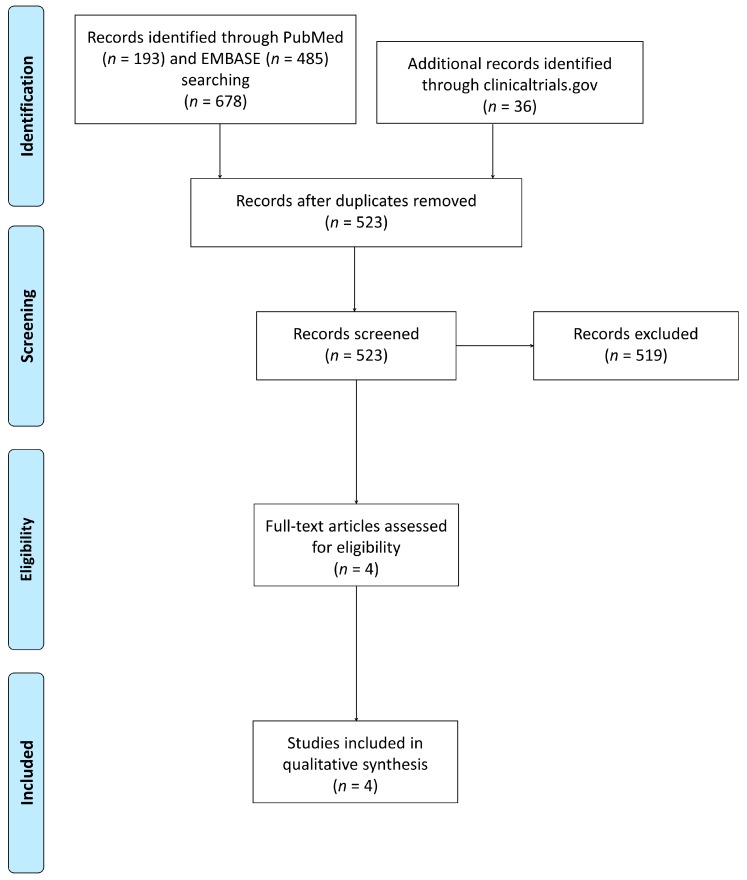
Flowchart of the study.

Authors found 678 articles using the described research strategies (193 articles in PubMed/Medline and 485 in Embase). Thirty-six clinical trials were identified in ClinicalTrials.gov. Duplicates were recognized and deleted using the EndNote program, reaching the number of 559 articles. Two authors were able to identify further duplicates performing a manual crosscheck using the names of authors, title and DOI number, reaching a final number of 523 articles (Figure 1). 

No randomized controlled studies were found applying the PICOS planned for this review. Results of this research did not find studies designed to compare two different cohorts of patients. However, authors found three retrospective observational studies and one prospective observational study which considered fibromyalgia among risk factors of postoperative surgical outcomes (Table 2). 

The study by Blonna et al. was aimed to retrospectively evaluate the prevalence of fibromyalgia in a cohort of consecutive patients attending the shoulder and elbow service of a single center [24]. Patients with a final diagnosis of fibromyalgia were 18 out of 286 (6.3%). These patients were subsequently asked to complete a shoulder questionnaire, the new Oxford shoulder (OS) score, a quality of life questionnaire, the short form-12 (SF-12), and a global Summary Outcome Determination score (SOD score). Authors found that, among the 18 patients finally diagnosed with fibromyalgia, only five had already received a diagnosis of fibromyalgia or received a diagnosis of fibromyalgia during the first appointment, with the remaining 13 patients diagnosed during one of the follow-up examinations. At the first evaluation, four patients received a diagnosis of rotator cuff tears, seven received a diagnosis of subacromial bursitis, one received a diagnosis of calcific tendinitis, and three received a diagnosis of adhesive capsulitis, whereas three patients had fibromyalgia as a single diagnosis. After an average follow-up time of 15 months, 56% of patients reported having severe symptoms with the OS score and 44% had mild to moderate symptoms.

Based on the SOD score, one patient stated that the shoulder was worse than before the treatment, 56% of patients reported that shoulder symptoms were unchanged, 28% reported some improvement, and 11% reported great improvement. None of the patients reported that the shoulder was normal or almost normal. Five patients received a total of 11 surgeries for shoulder pain, and among these, one patient was treated after the diagnosis of fibromyalgia was made. In the other four patients, fibromyalgia was misdiagnosed. All of these patients reported mild to severe symptoms at the last follow-up. 

Conclusions are that orthopedics should be aware that fibromyalgia could be the cause of failure in the treatment of concomitant painful shoulder [24]. 

The study by Cheng et al. was aimed to determine whether preoperative pain history or the fibromyalgia survey score could predict shoulder arthroscopy outcomes, such as pain and physical functioning and postoperative consumption of opioids [25].

One hundred shoulder arthroscopy patients completed preoperative validated self-reported measures of different variables such as pain and depressive symptoms and the fibromyalgia survey score, generated by the sum of the widespread pain index and symptom severity scale and ranging from 0 to 31 [9]. Outcomes were assessed on postoperative day two (opioid consumption, pain, physical functioning, and quality of recovery score) and 14 (opioid consumption and pain). Subjects were divided into tertiles for univariate analyses, which were referred to as “very low” (0–2), “low” (3–6), and “moderate” (7–13) groups. After multivariate modeling, authors observed that the fibromyalgia survey score was not associated with postoperative pain or opioid consumption. However, they found a significant correlation (*p* = 0.001) with the quality of recovery as assessed by the Quality of Recovery-9 scale after 2 days.

The study by Westermann et al. aimed to define opioid consumption and to evaluate patient factors that could be associated with prolonged opioid use after arthroscopy performed for rotator cuff repair (RCR) in the U.S. [26]. During the study period, 35,155 arthroscopic RCRs were studied. Authors reported that patients with fibromyalgia were more likely to be prescribed opioid medications after RCR, with a three-month follow-up risk ratio (RR) of 1.67 and a 95% confidence interval (CI) of 1.6–1.75, reaching a 12-month follow-up RR of 2.16 (95% CI: 2.03–2.31). Based on these results, authors recommended preoperative counseling of patients with this diagnosis.

The study by Rao et al. was based on the retrospective analysis of a large database with the goal of identifying patient risk factors for opioid use after shoulder arthroplasty [27]. The study sample consisted of 4243 shoulder arthroscopies performed in 3996 patients. Of these, 92 had a diagnosis of fibromyalgia (2.4% of the population). The primary outcome of the study was the number of dispensed opioid prescriptions for a post-operative follow-up of 360 days, divided in quarters of 90 days each: Q1, days 0–90; Q2, days 91–180; Q3, days 181–270; and Q4, days 271–360. Fibromyalgia was reported among the risk factors associated with higher opioid use during the Q4 follow-up (later rehabilitation period), with a reported incidence rate ratio of 1.20 (95% CI: 1.04–1.38).

The high heterogeneity among the included studies regarding study design and outcomes measured did not allow for a meta-analysis. Specific differences in study design and patient selection are summarized in Table 2 and the bias assessment is reported in Table 3.

## 4. Discussion

The results of this review demonstrate a severe lack of evidence regarding the influence of fibromyalgia in patients undergoing surgery for shoulder pain, without randomized or non-randomized controlled trials analyzing this aspect. Three retrospective observational studies and one prospective observational study were identified. Non-randomized observational studies in general provide a low quality of evidence [22]. Furthermore, none of the studies were designed to directly compare two different cohorts (patients with fibromyalgia syndrome vs. patients without fibromyalgia syndrome). Finally, the risk of bias due to several study limitations further reduces the quality of evidence. In particular, the study by Blonna et al. [24] is a retrospective study, and therefore baseline OS and SF-12 scores before treatment are not available; furthermore, only 5 patients with fibromyalgia underwent surgery, and therefore, sound conclusions regarding its influence on outcomes of surgery cannot be drawn. However, this is the first study exploring if fibromyalgia may have an impact on surgical outcomes in the shoulder and demonstrating that it is often misdiagnosed by orthopedics. The study by Cheng et al. [25] is the first to analyze fibromyalgia survey scores in a prospective design. However, it has several limitations, including the exclusion of patients who were already on chronic opioid therapy before surgery, the lack of an adjustment for confounding factors, and the very short follow-up periods (2 and 14 days), which limits the external validity of the results. In the study by Westermann et al. [26], the prevalence of fibromyalgia/myalgia patients in this study is around 22%, which is much higher than that in the other studies included in our systematic review. This is likely due to the fact that authors included patients with an International Classification of Diseases, Ninth Revision (ICD-9) code 729.1, which includes patients with a diagnosis of myalgia, fibromyalgia, or myositis. Classification criteria for fibromyalgia are not specified in the Methods section. Similarly, in the study by Rao et al. [27], no criteria for the diagnosis of fibromyalgia are reported, thus reducing the external validity of these results. Overall, our rate of confidence in the results of the studies included is low (Table 3).

Although the aim of our review was to find evidence on a very specific topic, i.e., the influence of fibromyalgia syndrome on surgical outcomes in patients with shoulder diseases requiring an orthopedic evaluation, the results are consistent with a lack of evidence. These findings do not necessarily mean a lack of correlation between worse surgical outcome and fibromyalgia syndrome. Indeed, we expect that a significant prevalence of fibromyalgia in patients referring to orthopedic surgeons for shoulder pain may emerge with specifically designed studies addressing this topic, based on the highly reported prevalence of fibromyalgia syndrome in the general population [3], on the evidence that the shoulder is among the most frequently affected regions in fibromyalgia syndrome [12], and on the evidence coming from lower-extremity joint replacement patients [15,18,28] and from other specialties such as gynecologic surgery [16,17].

## 5. Conclusions

The results of this systematic review demonstrate that limited evidence is currently available on the influence of fibromyalgia on clinical outcomes after shoulder surgery. Overall, patients with fibromyalgia undergoing surgery seem to be at risk of an increased use of analgesic drugs. The presence of a shoulder disease does not exclude the diagnosis of fibromyalgia, and orthopedic surgeons should be aware of this condition when approaching a patient with shoulder pain.

Future studies should be designed to specifically address the impact of fibromyalgia in shoulder surgical outcomes, evaluating postoperative pain and long-term follow-up with specific shoulder evaluation scales.

## Figures and Tables

**Table 1:** Fibromyalgia diagnostic criteria—2016 revision.

**Widespread Pain Index (WPI)**				
Left upper region (Region 1) □			Right upper region (Region 2) □	
□	Jaw *		□	
□	Shoulder girdle		□	
□	Upper arm		□	
□	Lower arm		□	
		
Left lower region (Region 3) □			Right lower region (Region 4) □	
□	Hip (buttock, trochanter)		□	
□	Upper leg		□	
□	Lower leg		□	
		
Axial region (Region 5)				
	Neck		□	
	Upper back		□	
	Lower back		□	
	Chest or breast *		□	
	Abdomen *		□	
Total score (0-19): ______				
**Symptom Severity Scale (SS) score**				
	0	1	2	3
Fatigue	□	□	□	□
Waking unrefreshed	□	□	□	□
Cognitive symptoms	□	□	□	□
	Present			
Headaches	□			
Pain/cramps in lower abdomen	□			
Depression	□			
Total score (0-12): ______				

For Widespread Pain Index (WPI): note the number of areas and the number of regions in which the patient has had pain over the last week. For the symptom severity scale (SSS) score: for each of the 3 symptoms (fatigue, waking unrefreshed, and cognitive symptoms) indicate the level of severity over the past week using the following scale: 0 = no problem; 1 = slight or mild problems, generally mild or intermittent; 2 = moderate, considerable problems, often present and/or at a moderate level; 3 = severe: pervasive, continuous, life-disturbing problems. SSS is the sum of the severity scores of the 3 symptoms (0–9) plus the sum (0–3) of the score of the presence of the following symptoms the patient refers over the past 6 months: (1) headaches; (2) pain or cramps in lower abdomen; (3) depression. Fibromyalgia severity scale is the sum of WPI and SSS. Criteria are satisfied if all conditions are met: (1) WPI ≥ 7 and SSS score ≥ 5 or WPI 4–6 and SSS score ≥ 9. (2) Generalized pain, defined as pain in at least 4 of 5 regions. (3) Symptoms generally present for at least 3 months. (4) A diagnosis of fibromyalgia does not exclude the presence of other clinically important illnesses. * Jaw, chest, and abdominal pain are not included in generalized pain definition.

**Table 2:** Summary and comparison of search results.

Author	Study Design	Sample Size	Type of Intervention	Outcomes	Conclusions	Follow-Up Time
Blonna et al. [24]	Retrospective observational	286 patients, including 18 with fibromyalgia, of which 5 underwent surgery (11 joints)	Orthopedic evaluation for shoulder pain and shoulder surgery	Diagnosis of fibromyalgia, new OSS, SF-12, and global SOD score	Fibromyalgia may be a cause of failure in the treatment of concomitant painful shoulder.	15 months (range: 12–27 months)
Cheng et al. [25]	Prospective observational	100 patients	Any type of shoulder arthroscopy	Opioid consumption, pain scores, neuropathic pain (PainDETECT), physical functioning (PROMIS), Quality of Recovery-9	A higher FSS does not correlate with postoperative opioid consumption, but with a lower 2nd-day postoperative Quality of Recovery-9 score.	14 days
Westermann et al. [26]	Retrospective case control	35,155 shoulder arthroscopies, including 7884 with myalgia or fibromyalgia	Arthroscopy for rotator cuff repair	Postoperative opioid prescriptions	Significantly more opioid prescriptions in fibromyalgia patients	12 months
Rao et al. [27]	Retrospective large database analysis	4243 surgery procedures in 3996 patients, including 92 with fibromyalgia	Elective shoulder arthroplasty	Postoperative opioid consumption	Higher opioid prescription in patients with fibromyalgia in the later rehabilitation period	360 days

FSS: fibromyalgia survey score, OSS: Oxford shoulder score, SF-12: short form-12; SOD: summary outcome determination.

**Table 3:** Newcastle–Ottawa assessment scale scores for the included studies.

Newcastle–Ottawa Quality Assessment Scale								
	Selection				Comparability	Outcome		
Author	Representativeness of the Exposed Cohort	Selection of the Non-Exposed Cohort	Ascertainment of Exposure	Demonstration that Outcome of Interest Was Not Present at the Start of Study	Comparability of Cohorts on the Basis of the Design or Analysis	Assessment of Outcome	Was Follow-Up Long Enough for Outcome to Occur?	Adequacy of Follow Up of Cohorts
Blonna et al. [24]	Truly representative ★	n/a	Surgical records ★	Yes ★	n/a	Self-reported outcomes and medical records ★	Yes ★	n/a
Cheng et al. [25]	Truly representative ★	n/a	Surgical records ★	Yes ★	n/a	Self-reported outcomes and medical records ★	No	n/a
Westermann et al. [26]	Fibromyalgia diagnosis based on ICD-9	n/a	Surgical records ★	Yes ★	n/a	Medical records ★	Yes ★	n/a
Rao et al. [27]	No description of fibromyalgia criteria	n/a	Surgical records ★	Yes ★	n/a	Medical records ★	Yes ★	n/a

ICD-9: International Classification of Diseases, Ninth Revision; n/a: not applicable because not studies comparing different cohorts.
